# Controlled Vapor-Phase Synthesis of VSe_2_ via Selenium-Driven Gradual Transformation of Single-Crystalline V_2_O_5_ Nanosheets

**DOI:** 10.3390/nano15070548

**Published:** 2025-04-04

**Authors:** Gangtae Jin

**Affiliations:** Department of Electronic Engineering, Gachon University, Seongnam 13120, Republic of Korea; gjin@gachon.ac.kr

**Keywords:** VSe_2_, V_2_O_5_, CVD, transformation

## Abstract

We report a gas-phase precursor modulation strategy for the controlled synthesis of 1T-phase vanadium diselenide (VSe_2_) from vanadium pentoxide (V_2_O_5_) nanosheets by systematically adjusting the vapor pressure of selenium. By controlling the selenium vapor pressure, selenium-free vapor transport of vanadium dioxide led to the spontaneous oxidation and formation of tens-of-micrometer-sized rectangular V_2_O_5_ crystals, while moderate selenium introduction produced intermediate oxygen-rich phases with trapezoidal crystal facets, and a highly selenium-rich environment yielded trigonal VSe_2_ crystals. Raman scattering measurements confirmed the stepwise transformation from V_2_O_5_ to VSe_2_, and atomic force microscopy revealed well-defined layered morphologies and distinct conformation within an atomically thin regime. Additionally, high-resolution transmission electron microscopy validated the orthorhombic and trigonal crystal structures of V_2_O_5_ and VSe_2_, respectively. This work demonstrates the versatility of fine-tuned vapor-phase growth conditions in vanadium-based layered compounds, providing useful platforms to optimize structural composition with atomic precision.

## 1. Introduction

Nanostructured transition metal dichalcogenides (TMDCs) with two-dimensional (2D) layered structures have attracted substantial interest due to their unique electronic, optical, and catalytic properties. These materials exhibit exotic phenomena, such as charge density waves (CDWs), superconductivity, and phase transitions, making them attractive for future computing applications, including field-effect transistors, optoelectronic devices, and energy storage systems [1,2,3,4,5,6,7,8,9,10,11,12,13,14,15,16]. Among TMDCs, vanadium diselenide (VSe_2_) stands out because of its intriguing physical properties. In its 1T phase, VSe_2_ is metallic and exhibits a CDW transition at low temperatures below ~110 K. Despite the promises for potential applications such as in memristor and neuromorphic hardware, the electronic structure of 1T VSe_2_ is often sensitive to external factors such as air exposure and point defects, requiring thorough understanding of its synthesis protocol with controlled stoichiometry and composition [17,18,19,20]. Meanwhile, vanadium pentoxide (V_2_O_5_), a dissimilar layered vanadium compound, is widely utilized in electrochemical energy storage, catalysis, and optoelectronics [21,22,23,24,25,26,27,28]. It possesses a stable orthorhombic crystal structure and a high oxidation state of vanadium, making it a suitable template for transformation into lower-oxidation-state vanadium chalcogenides [29,30,31]. The precision transformation from atomically thin V_2_O_5_ to VSe_2_ involves the reduction of vanadium and the incorporation of selenium, leading to versatile material platforms.

Here, we demonstrated a vapor-phase synthesis approach for VSe_2_ through the selenium-induced transformation of single-crystalline V_2_O_5_ nanosheets. By precisely tuning the selenium vapor pressure, we directed the stepwise transformation from V_2_O_5_ to VSe_2_ via intermediate oxygen-rich vanadium selenide phases. Our findings offer insights into the role of precursor modulation in TMDC growth and pave the way for designing high-quality layered materials with tailored electronic properties.

## 2. Results and Discussion

### 2.1. Vapor-Phase Control of Selenium for Synthesis of O-Rich and Se-Rich Crystals

**Figure 1:**
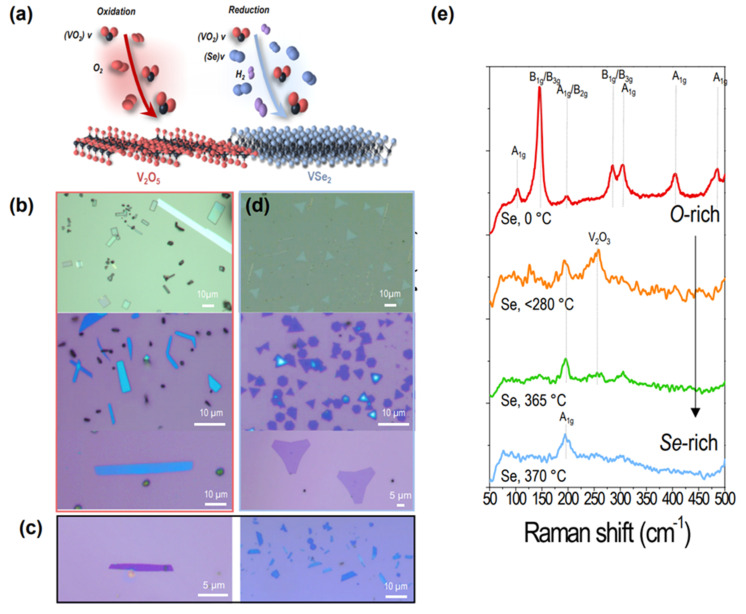
(**a**) Growth schematics of single-crystalline orthorhombic V_2_O_5_ and trigonal VSe_2_ nanosheets. (**b**) Optical microscope images of layered V_2_O_5_ on sapphire substrate (top) and SiO_2_ (bottom). (**c**,**d**) Optical microscope images of O-rich intermediate phases with trapezoidal-faceted crystals (**c**) and trigonal VSe_2_ (**d**) on sapphire substrate (top) and SiO_2_ (bottom). (**e**) Normalized Raman spectra of V_2_O_5_ (selenium at 0 °C, red), O-rich VSe_2_ (selenium at <280 °C, orange), Se-rich VSe_2_ (selenium at 365 °C, green), and VSe_2_ (selenium at 370 °C, blue). a.u., arbitrary unit.

The controlled synthesis of VSe_2_ was achieved by progressive precursor modulation, increasing the vapor pressure of selenium and maintaining that of VO_2_ (Figure 1a). First, in the absence of selenium, rutile VO_2_ was synthesized in a chemical vapor deposition system. Layered V_2_O_5_ nanosheets with a lateral size of tens of micrometers were grown by the spontaneous oxidation of VO_2_ vapor, forming rectangular-faceted single crystals (Figure 1b), presumably due to diffusion-limited growth, where oxygen diffusion to the surface controlled the rate of oxidation. Ar/H_2_ mixture gas also acted as a reductant, helping to reduce VO_2_ towards a lower oxidation state. Then, the selenium-driven transformation of V_2_O_5_ nanosheets was achieved by introducing the vapor pressure of selenium (280~370 °C) and maintaining other growth parameters (total pressure, amount of precursor powder, flow rate of Ar/H_2_) during the reactions, resulting in the gradual modification of facets from rectangles to trapezoids, as shown in Figure 1c. We identified these crystals as intermediate states between V_2_O_5_ and VSe_2_. Under the selenium-rich environment (selenium at 370 °C), trigonal VSe_2_ crystals revealed triangular facets on sapphire substrate (top) and SiO_2_ substrates (bottom) (Figure 1d), highlighting a complete transformation. The gradual transformation was may attributed to the vapor pressure of selenium gradually substituting the oxygen atoms within the lattice, thereby modifying the vanadium oxidation state. This gradual transformation from V_2_O_5_ to VSe_2_ was verified by Raman characterization. To confirm the gradual transformation with consistency, Raman scattering spectra (Figure 1d) were measured from four individual batches. With considerable Se addition, a dramatic change in the peak positions was also identified via Raman spectroscopy (Figure 1e and Table 1). Under the growth condition without selenium vapor, which was not heated in a separate zone, the rectangular crystal showed seven prominent scattering peaks [103.7 cm^−1^ (A_1g_), 145.6, 284.7 cm^−1^ (B_1g_/B_3g_), 196.8 cm^−1^ (A_1g_/A_2g_), 304.4, 405.4, and 484.1 cm^−1^ (A_1g_)]. Under the condition with selenium at <280 °C, those peaks were not noticeable, showing new peaks at ~255 cm^−1^, which was attributed to V_2_O_3_, and 195 cm^−1^, indicative of an intermediate selenization stage. Sufficient amounts of Se at 365~370 °C resulted in a clear peak of A_1g_ (200.6 cm^−1^) vibration modes, confirming the formation of trigonal VSe_2_.

### 2.2. Material Characterization of Atomically Thin VSe_2_ and V_2_O_5_ Nanosheets

**Figure 2:**
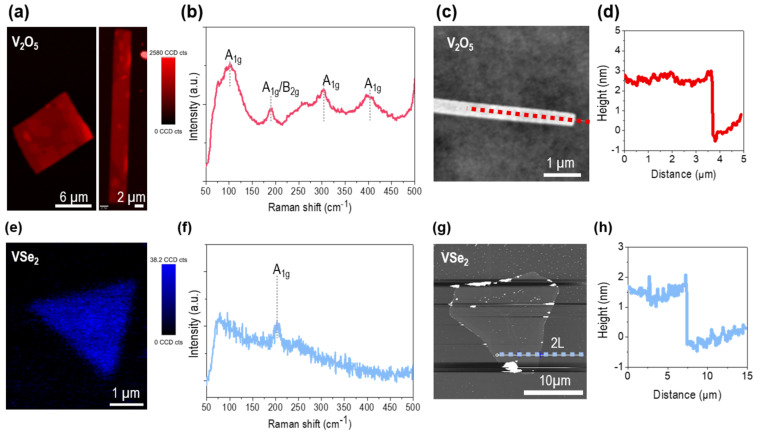
(**a**,**b**) Raman mapping image (**a**) and representative spectrum (**b**) of V_2_O_5_ crystals. (**c**,**d**) AFM image (**c**) and corresponding height profile (**d**) of V_2_O_5_ crystal obtained from dotted line. (**e**,**f**) Raman mapping image (**e**) and representative spectrum (**f**) of VSe_2_ crystals. (**g**,**h**) AFM image (**g**) and corresponding height profile (**h**) of VSe_2_ crystal obtained from dotted line. a.u., arbitrary unit.

Se-rich and O-rich vanadium compounds were further optimized to thin down the thickness of the crystals. Alkali halide (potassium iodide) was introduced to act as a nucleation inhibitor to suppress the nucleation density. The alkali halide is known for its function as a surfactant, reducing surface energy and thereby limiting excessive nucleation events. To characterize the atomically thin nanosheets of VSe_2_ and V_2_O_5_ obtained under two different conditions using the controlled vaporization of selenium, we performed Raman mapping at a wavelength of 532 nm. In Figure 1a, a rectangular crystal with additional vertically overgrown layers is revealed in the Raman mapping images. This crystal showed four Raman peaks (102.3 cm^−1^, 190.2 cm^−1^, 303.2 cm^−1^, and 401.5 cm^−1^), as shown in Figure 2b, which were partially red-shifted, as indexed in Table 1 [32,33]. These V_2_O_5_ nanosheets were characterized by atomic force microscopy (Figure 2c), displaying clean surface and edges in the AFM scan images even at a thickness of 3 nm. Atomically thin VSe_2_ showed weak intensities in the Raman mapping image (Figure 2e). It had a sole vibration mode at ~206 cm^−1^ that was blue-shifted, indicating a reduced layer thickness compared to the reference bulk crystals. The surface of VSe_2_ was contaminated, as we observed oxidized chunks at the crystal edges in the AFM mapping image with the step height of a bilayer.

### 2.3. Crystal Structure of Orthorhombic V_2_O_5_ and Trigonal VSe_2_

**Figure 3:**
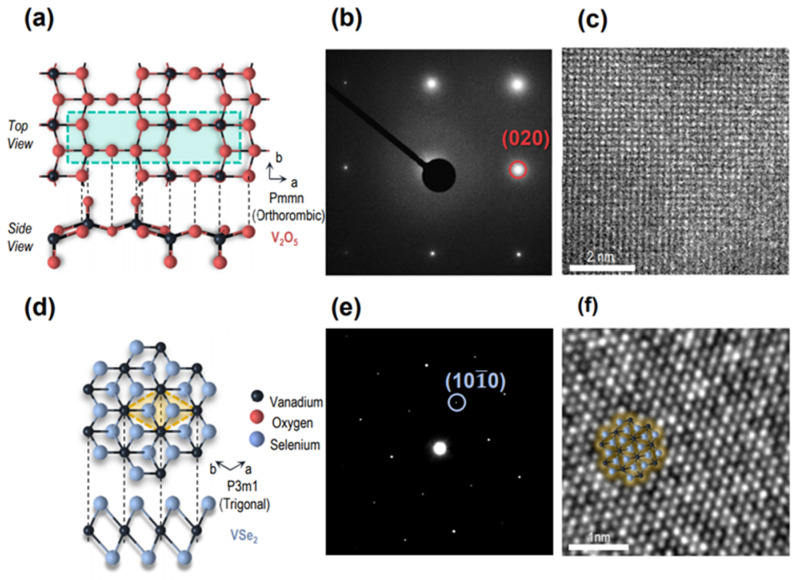
(**a**) Crystal structure of orthorhombic V_2_O_5_. (**b**) Selected area electron diffraction (SAED) pattern collected from V_2_O_5_ on thin amorphous SiO_2_ layer. (**c**) High-magnification TEM image of V_2_O_5_ specimen. (**d**) Crystal structure of trigonal VSe_2_. (**e**) SAED pattern collected from VSe_2_. (**f**) High-magnification TEM image of few-layer VSe_2_ crystal.

The atomic structures of the VSe_2_ and V_2_O_5_ were characterized by transmission electron microscopy (TEM). Due to the inability to transfer V_2_O_5_ grown on SiO_2_ substrates using hydrofluoric acid or potassium hydroperoxide etching, the focused ion beam (FIB) was exploited to pick up V_2_O_5_/SiO_2_ stacks. The prepared V_2_O_5_ specimen had an orthorhombic crystal structure, which was classified as a *Pmmn* space group, as shown in Figure 3a. The plan-view V_2_O_5_ showed rectangular atomic arrangements with corresponding (020) diffraction spots, as shown in the selected-area electron diffraction (SAED) pattern in Figure 3b,c, although vanadium atoms were only attributed to this TEM image. Additionally, high-resolution bright-field TEM indicated that VSe_2_ multilayers (Figure 3d) exhibited a trigonal structure with (10-10) diffraction spots (Figure 3e,f) despite the substantial contamination along the edges of the crystal that was observed in the AFM scan image [20]. The observed diffraction patterns confirmed the integrity of the synthesized layers, with no detectable alloying effects between VSe_2_ and V_2_O_5_.

## 3. Materials and Methods

### 3.1. Chemical Vapor Deposition (CVD) of Atomically Thin Vanadium Compounds

The synthesis of V_2_O_5_ and VSe_2_ was carried out using a CVD approach. V_2_O_5_ nanosheets were synthesized via the vapor-phase oxidation of vanadium dioxide (VO_2_). High-purity VO_2_ powder (99.99%) was placed in a ceramic boat at the center of a 3 in. quartz tube furnace, and a 1 cm × 1 cm sapphire or 2 cm × 2 cm SiO_2_/Si substrate was positioned downstream with a 1–1.5 cm gap from the solid powder. The furnace was heated to 850 °C under an Ar/H_2_ (20:15 sccm) flow and maintained for 5 min, promoting the spontaneous oxidation of VO_2_ into layered V_2_O_5_ nanosheets. The furnace was naturally cooled down to room temperature. The chamber pressure was held around 600–700 torr. For VSe_2_, selenium vapor was additionally introduced to generate a product of VSe_2_. Selenium powder (99.99%) was separately heated to temperatures ranging around 370 °C to control the vapor pressure. The reaction proceeded at 820 °C under a reducing atmosphere of Ar/H_2_ (23:11 sccm) for a duration of 5 min, enabling a gradual substitution of oxygen with selenium. The chamber pressure was held around 600–700 torr.

### 3.2. Gradual Selenium Control Mechanism

To investigate the effect of selenium’s vapor pressure, we performed a series of reactions with varying selenium source temperatures. At lower temperatures (<280 °C), selenium diffusion was limited, leading to the formation of oxygen-rich intermediate phases. These structures exhibited trapezoidal crystal facets, indicative of an incomplete conversion process. At moderate temperatures (280–365 °C) in the selenium region, increased selenium vapor pressure facilitated a higher degree of substitution, forming partially selenized vanadium oxyselenide compounds. At a selenium temperature of 370 °C, a complete phase transition to trigonal VSe_2_ was achieved.

## 4. Conclusions

In summary, we have demonstrated a controlled vapor-phase synthesis strategy for VSe_2_ via the selenium-driven transformation of atomically thin V_2_O_5_ nanosheets. By precisely tuning the vapor pressure of selenium, we directed a stepwise transition from V_2_O_5_ nanosheets to VSe_2_ nanosheets through intermediate phases of oxygen-rich vanadium selenides. Comprehensive structural characterization using Raman spectroscopy, atomic force microscopy, and high-resolution transmission electron microscopy confirmed the phase transformation, layered morphology, and high crystallinity of the synthesized materials. This work highlights the importance of vapor phase precursor modulation in tailoring phase transitions within vanadium-based layered chalcogenides, offering a versatile platform for tuning electronic properties at the nanoscale. Potentially, similar vapor-phase modulation methods could be extended to other vanadium-based chalcogenides, such as VS_2_ or VTe_2_. The insights provide further advancements in the precision synthesis and integration of VSe_2_ for future electronic, optoelectronic, and neuromorphic computing applications.

## Figures and Tables

**Table 1:** Summary of Raman peaks and related vibration modes at 532 nm laser excitation.

Sample	Raman Shift (cm^−1^)	Vibration Mode
VSe_2_	200.3	A_1g_
2L-VSe_2_	206.1	A_1g_
V_2_O_5_	103.7	A_1g_
V_2_O_5_	145.6	B_1g_/B_3g_
V_2_O_5_	196.8	A_1g_/B_2g_
V_2_O_5_	284.7	B_1g_/B_3g_
V_2_O_5_	304.4	A_1g_
V_2_O_5_	405.4	A_1g_
V_2_O_5_	484.1	A_1g_
3 nm thick V_2_O_5_	102.3	A_1g_
3 nm thick V_2_O_5_	190.2	A_1g_/B_2g_
3 nm thick V_2_O_5_	303.2	A_1g_
3 nm thick V_2_O_5_	401.5	A_1g_

## Data Availability

The original contributions presented in this study are included in the article. Further inquiries can be directed to the corresponding author.
